# The clinical benefit of array-based comparative genomic hybridization for detection of copy number variants in Czech children with intellectual disability and developmental delay

**DOI:** 10.1186/s12920-019-0559-7

**Published:** 2019-07-23

**Authors:** Marketa Wayhelova, Jan Smetana, Vladimira Vallova, Eva Hladilkova, Hana Filkova, Marta Hanakova, Marcela Vilemova, Petra Nikolova, Barbora Gromesova, Renata Gaillyova, Petr Kuglik

**Affiliations:** 1 Institute of Experimental Biology, Faculty of Science, Masaryk University, Kotlarska 267/2, Brno, Czech Republic; 2 Department of Medical Genetics, University Hospital Brno, Cernopolni 212/9, Brno, Czech Republic

**Keywords:** Intellectual disability, Developmental delay, Microdeletion, Microduplication, CNV, Array-CGH

## Abstract

**Background:**

Chromosomal microarray analysis has been shown to be a valuable and cost effective assay for elucidating copy number variants (CNVs) in children with intellectual disability and developmental delay (ID/DD).

**Methods:**

In our study, we performed array-based comparative genomic hybridization (array-CGH) analysis using oligonucleotide-based platforms in 542 Czech patients with ID/DD, autism spectrum disorders and multiple congenital abnormalities. Prior to the array-CGH analysis, all the patients were first examined karyotypically using G-banding. The presence of CNVs and their putative derivation was confirmed using fluorescence in situ hybridization (FISH), multiplex ligation-dependent probe amplification (MLPA) and predominantly relative quantitative polymerase chain reaction (qPCR).

**Results:**

In total, 5.9% (32/542) patients were positive for karyotypic abnormalities. Pathogenic/likely pathogenic CNVs were identified in 17.7% of them (96/542), variants of uncertain significance (VOUS) were detected in 4.8% (26/542) and likely benign CNVs in 9.2% of cases (50/542). We identified 6.6% (36/542) patients with known recurrent microdeletion (24 cases) and microduplication (12 cases) syndromes, as well as 4.8% (26/542) patients with non-recurrent rare microdeletions (21 cases) and microduplications (5 cases). In the group of patients with submicroscopic pathogenic/likely pathogenic CNVs (13.3%; 68/510) we identified 91.2% (62/68) patients with one CNV, 5.9% (4/68) patients with two likely independent CNVs and 2.9% (2/68) patients with two CNVs resulting from cryptic unbalanced translocations. Of all detected CNVs, 21% (31/147) had a de novo origin, 51% (75/147) were inherited and 28% (41/147) of unknown origin.

In our cohort pathogenic/likely pathogenic microdeletions were more frequent than microduplications (69%; 51/74 vs. 31%; 23/74) ranging in size from 0.395 Mb to 10.676 Mb (microdeletions) and 0.544 Mb to 8.156 Mb (microduplications), but their sizes were not significantly different (*P* = 0.83). The pathogenic/likely pathogenic CNVs (median 2.663 Mb) were significantly larger than benign CNVs (median 0.394 Mb) (*P* < 0.00001) and likewise the pathogenic/likely pathogenic CNVs (median 2.663 Mb) were significantly larger in size than VOUS (median 0.469 Mb) (*P* < 0.00001).

**Conclusions:**

Our results confirm the benefit of array-CGH in the current clinical genetic diagnostics leading to identification of the genetic cause of ID/DD in affected children.

**Electronic supplementary material:**

The online version of this article (10.1186/s12920-019-0559-7) contains supplementary material, which is available to authorized users.

## Background

Intellectual disability and developmental delay (ID/DD) represent a worldwide health and social problem with a prevalence of 1–3% in children from Western European population. These patients are characterized by significantly reduced abilities to understand new or complex information and to learn and apply new skills, resulting in a limited ability to cope independently. The deficiencies manifest before the 18th year, with lasting effect on development [1]. Most children with mild ID are diagnosed in early school age because of developmental delay and developmental disorders, while children with severe forms of ID can receive their clinical diagnose earlier, within the first 2 years of life [2]. ID/DD can occur as part of clinically defined rare syndromes in combination with multiple congenital anomalies (MCA) or other neurological and neurodevelopmental features as epilepsy, sensory disorders or autism spectrum disorders (ASD) with highly variable severity (syndromic ID/DD), while non-syndromic ID/DD patients typically manifest ID/DD as a single clinical feature [3]. In clinical practice, these categories often merge due to less apparent dysmorphic or other abnormal features which may be overlooked. The genetic background of ID/DD and ASD is recognised as the most common identifiable cause in their pathogenic progression. Recent worldwide studies frequently demonstrate extreme genetic heterogeneity in the pathogenesis of ID/DD and ASD varying from large chromosomal abnormalities to point mutations in single genes that have been documented in a wide spectrum of copy-number variants (CNVs), single gene sequence variants or small insertions, deletions or duplications in DNA sequence or defects in the epigenetic regulation of gene expression [3–5].

In current genetic diagnostics, the method of array-based comparative genomic hybridization (array-CGH) has become a routine analytical technique for the detection of CNVs in patients with ID/DD, ASD and MCA. In general, there are two major groups of CNVs: recurrent and non-recurrent CNVs. Briefly, recurrent CNVs arise following non-allelic homologous recombination (NAHR) during meiosis between low-copy repeats encompassing large gene-rich blocks as reviewed by Weise et al. (2012) and Watson et al. (2014) [6, 7]. This model is evidenced by the occurrence of breakpoints in the same or a very similar genomic position (allowing for differences between the resolutions of microarray platforms) in patients with recurrent microdeletions/microduplications. Conversely, non-recurrent unique CNVs arise from different events that do not depend directly on genomic architecture, and include non-homologous end joining (NHEJ) and processes of fork stalling and template switching (FoSTeS) [8–10]. Recent studies on large cohorts show that submicroscopic chromosomal rearrangements can explain the pathological phenotype in about 15–20% children with ID/DD [11, 12]. Due to its high sensitivity, accuracy and diagnostic yield, array-CGH is now recommended as a first-tier test for ID/DD [12, 13]. In the past, array-CGH led to identification of multiple microdeletion/microduplication syndromes and still facilitates the detection and characterization of novel, cryptic submicroscopic, non-recurrent chromosomal rearrangements. Although “next-generation” sequencing (NGS) is now rapidly evolving as the ultimate approach to molecular genetic diagnostics of children with ID/DD, array-CGH still represents widespread gold standard in the diagnostic practice.

In this study we report the diagnostic yield of pathogenic/likely pathogenic recurrent and non-recurrent CNVs on a cohort of 542 Czech children with ID/DD, ASD and MCA. Simultaneously we demonstrate the usefulness of array-CGH as a clinical test in the genetic counselling and evolving challenges in the data interpretation.

## Methods

A total of 542 children with ID/DD, ASD and MCA were diagnosed at the Department of Medical Genetics (University Hospital Brno, Czech Republic) in the period from 2012 to 2017, including 215 girls and 327 boys with median age of 5 years (0–18 years). Almost 60% (315/542) were examined before the age of six years (0–5 years) and only 5% (28/542) were older than 15 years (16–18 years) at the time of array-CGH analysis.

Prior to any genetic analysis being performed, parents signed an informed consent. All patients first received standard karyotyping using a G-banding procedure. The G-banded karyotypes were analysed, documented and archived for each patient using the LUCIA Cytogenetics™ image analysis system (Laboratory Imaging, Prague, Czech Republic). Genomic DNA samples were obtained from leukocytes of peripheral blood in EDTA according to the standard DNA isolation process using MagNaPure system (Roche Ltd., Basel, Switzerland). Their quality and quantity were assessed by NanoDrop® ND-1000 (ThermoFisher Scientific, Waltham, MA, USA) and Qubit® 1.0 (ThermoFisher Scientific).

The whole-genomic screening of chromosomal rearrangements by array-CGH was performed using DNA microarray platforms (180 K and 60 K): SurePrint G3 Human CGH + SNP Microarray 4x180K, SurePrint G3 Human CGH Microarray 4x180K, SurePrint G3 Human CGH Microarray 8x60K (Agilent Technologies, Santa Clara, CA, USA) and Cytosure ISCA 4X180K UPD array (Oxford Gene Technology, Oxfordshire, UK) according to the manufacturers’ recommendations. The technical characteristics of all microarray platforms are available on manufacturer’s websites. All samples were matched with Human Genomic DNA reference (Agilent Technologies or Promega, Madison, WI, USA). Microarray slides were scanned with a DNA Microarray Scanner (Agilent Technologies). Data were obtained using the Agilent Feature Extraction software 12.0.2.2 and Agilent CytoGenomics 4.0, respectively, and visually checked using the Agilent Genomic Workbench Software 7.0.4.0 and Agilent CytoGenomics 4.0. CNVs were detected using the ADM-2 algorithm with filters of minimal size of 200 kb in region, > 5 Mb of copy-number neutral loss of heterozygosity (cnnLOH) regions (in CGH + SNP analyses), and a minimal absolute average log ratio of 0.25 as cut-off. Genomic positions were estimated using the human genomic reference sequence GRCh37/hg19 when using the DNA microarray platforms of Agilent Technologies, and the reference sequence GRCh36/hg18 when employing DNA microarray platforms by Oxford Gene Technology (OGT).

### CNV analysis and classification

For clinical use, every detected non-polymorphic CNV requires interpretation. As outlined elsewhere [14, 15], we considered a set of minimal criteria to interpret CNVs as 1) pathogenic or likely pathogenic CNVs, 2) variants of uncertain significance (VOUS), 3) likely benign CNVs and 4) variants with insufficient evidence for their clinical significance (not assessed). We followed the decision tree as presented by Buysse et al. (2009) [16]. The two-tailed Mann–Whitney U test was used to compare CNVs sizes, the test was run at the 5% level of significance.

### CNV confirmatory testing

#### FISH and MLPA analysis

Fluorescence in situ hybridization (FISH) and multiplex ligation-dependent probe amplification (MLPA) were used to confirm the CNV findings and establish the inheritance, as described elsewhere [17–19]. The confirmatory method was selected according to availability of parental genetic material and perceived clinical value of the given method.

#### Relative quantitative PCR (qPCR)

Relative qPCR was performed with two pairs of DNA primers which were designed to prime both the inside and outside of CNVs, and one pair of DNA primers targeting the *ERH* gene as an endogenous control. The reactions were run in duplicates, following the manufacturer’s recommendations (ThermoFisher Scientific). C_T_ values were subtracted for the *ERH* gene and each tested DNA region, for which was evaluated to derive R-values. An R-value < 0.7 for DNA loss and > 1.3 for DNA gain in the targeted DNA regions relatively to the *ERH* gene were set as cut-offs. The DNA primers were synthesized using a commercially available service (Integrated DNA Technologies, Coralville, Iowa, USA).

## Results

In the 6-year period from 2012 to 2017, a total of 542 children with ID/DD, MCA and ASD were examined according to afore mentioned multi-step molecular genetic diagnostic procedure at the Department of Medical Genetics (University Hospital Brno). First their karyotype was examined by G-banding followed by whole-genomic screening of CNVs by microarray. Using array-CGH, 32.7% of them (177/542) exhibited non-polymorphic CNVs or large regions of cnnLOH.

Overall, we identified clinically relevant CNVs for ID/DD and associated comorbidities in 17.7% of patients (96/542) which were classified as pathogenic or likely pathogenic CNVs, the VOUS were detected in 4.8% of cases (26/542) and benign CNVs in 9.2% of cases (50/542). Among all 542 cases, 5.9% patients had initial cytogenetic abnormalities (32/542) which were subsequently confirmed and specified by array-CGH (100%; 32/32) (see Additional file 1).

**Fig. 1:**
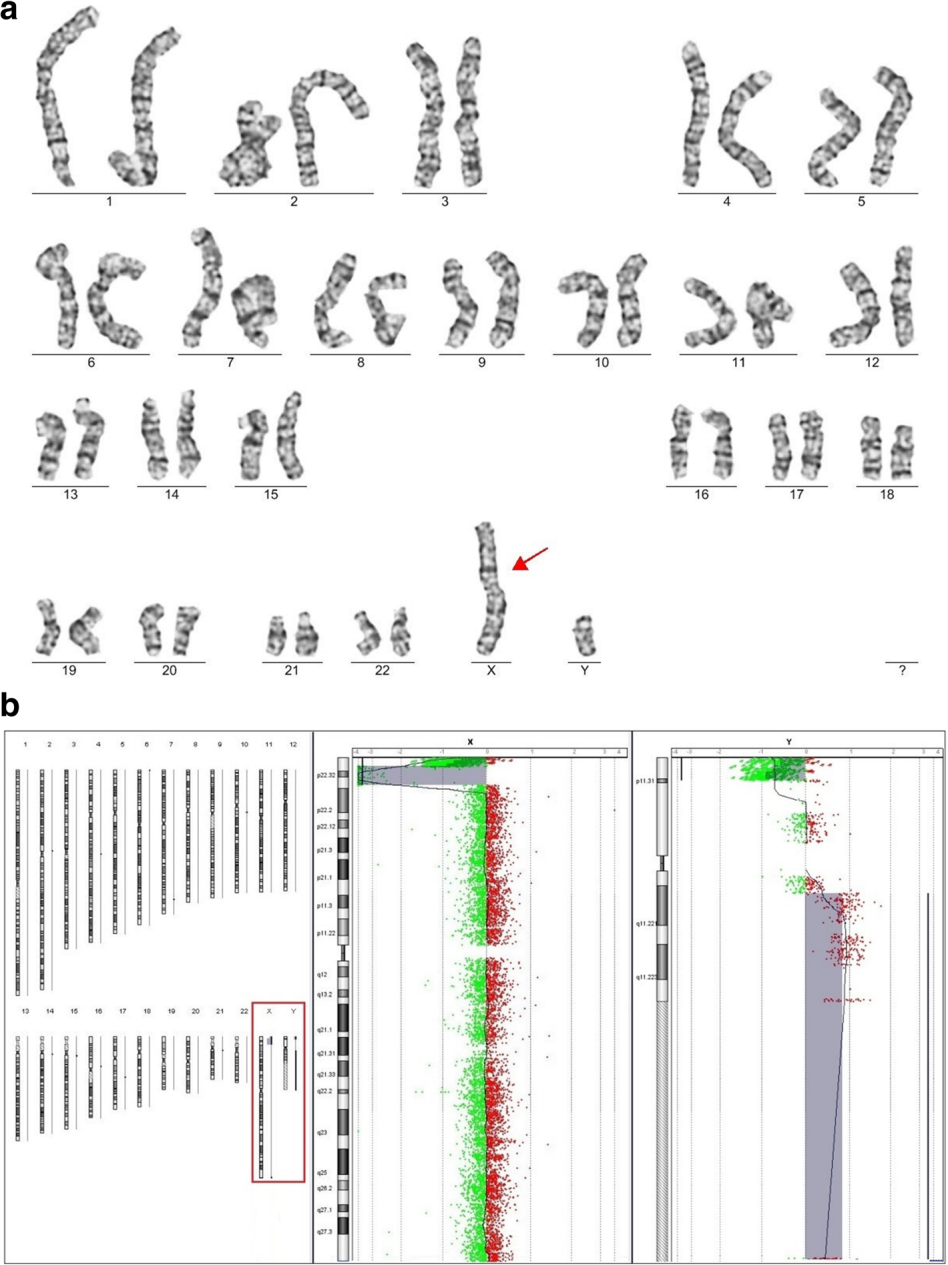
The example of a patient with the cytogenetic finding in the karyotype and array-CGH profile with specification of this chromosomal aberration. **a** The cytogenetic analysis of karyotype: 46,der(X)t(X;Y),Y. The 2-year-old male patient with developmental delay and congenital malformations of genital organs has a karyotype 46,der(X)t(X;Y),Y and array-CGH has confirmed and specified this chromosomal aberration. **b** The array-CGH profile presenting array-CGH result: arr [GRCh37] Xp22.33p22.31(2701273_8531171)× 0,Yq11.221q12(15991426_59002403)× 2. The 2-year-old male patient with developmental delay and congenital malformations of genital organs has a karyotype 46,der(X)t(X;Y),Y and array-CGH has confirmed and specified this chromosomal aberration

An example of a positive cytogenetic finding in karyotype that was subsequently confirmed by array-CGH (patient 280) is presented in the Fig. 1a, b.

The 2-year-old male patient with developmental delay and congenital malformations of his genital organs had: a) the karyotype analysis identified an unbalanced translocation t(X;Y) and the presence of normal Y chromosome. The derived chromosome X was inherited from an affected mother with a karyotype 46,X,t(X;Y)(p22.3;q11.21).

b) The array-CGH analysis identified a Xp22.31-p22.32 microdeletion (5.83 Mb) and Yq11.221-q12 duplication (43.01 Mb). The patient has no copy of Xp22.31-p22.32 locus and two copies of Yq11.221-q12 locus. Combining both karyotype and array-CGH analyses, the Yq11.221-q12 part is translocated to the short arm of X chromosome lacking Xp22.31-p22.32 locus.

### The findings of single pathogenic/likely pathogenic CNV

Considering 510 patients with normal karyotype, we detected submicroscopic pathogenic or likely pathogenic CNVs in 13.3% patients (68/510). In 12.2% (62/510) one clinically relevant CNV was identified. The group included 24 patients diagnosed with recurrent microdeletion syndromes: 1q21.1-q21.2 (3x), 1q43-q44 (3x), 7q11.23 (1x), 15q11.2 (3x), 15q1.1-q13.1 (1x), 15q13.2-q13.3 (1x), 16p11.2 (3x), 16p12.2 (1x), 16p13.11 (1x), 17p11.2 (1x), 17q12 (1x), 22q11.21 or distal 22q11.21-q11.22 (4x) and 22q13.3 (1x). Also, 12 patients were diagnosed with recurrent microduplication syndromes: 1q21.1-q21.2 (2x), 15q11.2-q13.1 (3x), 16p11.2 (1x), 17q11.2 (2x), 17q12 (1x) and 22q11.21 (3x) (see Additional file 2). The remaining 26 cases were represented by non-recurrent microdeletion (21 cases) and microduplication CNVs (5 cases) (see Additional file 3).

### The findings of two pathogenic/likely pathogenic CNVs (microdeletion/microduplication)

In four cases the two likely independent pathogenic/likely pathogenic CNVs were detected. In patient 151, we identified a 2q12.1-q12.3 microduplication (3.411 Mb) and 2q13 microduplication (1.802 Mb). Parental DNA samples were not available to specify the origin of these CNVs. In the case of patient 274, we identified a 2q23.1-q23.3 microdeletion (4.922 Mb) and 7q21.12-q21.13 microduplication (1.682 Mb). In patient 21, we identified maternally inherited 4q12-q13.1 (10.677 Mb) and 9q21.1 (0.813 Mb) microdeletions with array-CGH. In the case of patient 451, we identified a de novo terminal 10q26.2-q26.3 microdeletion (6.231 Mb) and maternally inherited interstitial 11p14.3-p15.1 microduplication (6.667 Mb).

Two other patients carried two pathogenic CNVs resulting from cryptic unbalanced translocations. In both cases, each patient was shown to carry one terminal microdeletion and one terminal microduplication indicating familial occurrence of a balanced translocation.

In the case of patient 380, we detected a terminal 7q36.1-q36.2 microdeletion (6.827 Mb) and terminal 12p13.31-p13.33 microduplication (6.324 Mb): subtelomeric metaphase FISH showed the cryptic balanced translocation t (7;12)(q36.3;p13.33) to be of paternal origin.

Patient 430 was a female with a 46,XY karyotype. Using array-CGH we further detected a terminal 9p23-p24.3 microdeletion (9.832 Mb) and terminal 16q23.3-q24.3 microduplication (8.156 Mb) in which the size of both CNVs was at the resolution limit of classical cytogenetics. To date, the origin of these CNVs has not been assessed. The summaries of patients with two pathogenic/likely pathogenic CNVs are presented in Additional file 4.

### Classification and characteristics of CNVs

Considering only the cases with pathogenic or likely pathogenic submicroscopic chromosomal rearrangements, microdeletions were more frequent than microduplications (69%; 51/74 vs. 31%; 23/74), however, their sizes (median 2.547 Mb for microdeletions, range 0.395–10.676 Mb; median 2.823 Mb for microduplications, range 0.544–8.156 Mb) did not differ significantly (*P* = 0.83).

Also, 4.8% of all array-CGH findings (26/542) were classified as VOUS. In detail, we identified total of 17 structural CNVs with uncertain significance in 15 patients, 3 cases presented only gonosomal aneuploidies (47,XXX in two cases and 47, XYY in one case) and 8 cases with large regions of cnnLOH > 5 Mb. These outputs are presented in the Additional file 5. In general, the structural VOUS were typically gene-poor: 7 VOUS did not contain any disease-related OMIM genes, 6 VOUS contained per 1 disease-related gene, 1 VOUS contained 2 disease-related genes and 2 VOUS contained per 5 disease-related genes. Focusing on genotype-phenotype correlations the losses or gains of these genes did not fully explain the phenotypic abnormalities in our patients. In one case we detected maternally derived marker chromosome 21 (6.032 Mb), containing 2 disease-related OMIM genes. The size of remaining 16 submicroscopic VOUS varied from 0.24 Mb to 3.238 Mb and microduplications were 3-fold frequent than microdeletions (12 vs. 4).

Using CGH + SNP array platform we detected 8 cases of large cnnLOH regions. In 6 cases the children were from consanguineous families so we observed more than one cnnLOH locus across their genomes. The remaining cases did not have a family history of consanguinity. These results providing some important information on the genomic location, size and gene content were essential for additional molecular genetic analyses by Sanger sequencing e.g. in patient 10 from consanguineous family. We identified cnnLOH > 5 Mb in chromosomal loci 4q28.3, 10q11.22q21.2, 11q22.1q22.3 and 12q24.23q24.32 and analysed their gene content (see Additional file 5). Based on her phenotypic features of suspected Cockayne syndrome we performed Sanger sequencing of the *ERCC6* gene which is located in the chromosomal locus 10q11.23. We detected a nonsense sequence variant c.3259C > T (p.Arg1087X) in homozygous state and confirmed the diagnosis on molecular genetic level (data in the medical record).

The CNVs in 9.2% cases were classified as likely benign with 57 CNVs in 50 patients (50/542). To summarize, the likely benign CNVs were gene-poor, only 29.8% of them (17/57) contained any disease-related OMIM gene or genes (see Additional file 6). In those cases, the gene content did not correlate with the patient’s phenotype and 88.2% (15/17) these CNVs were proved to be inherited from normal parent. The size of likely benign CNVs varied from 0.205 Mb to 1.469 Mb and microduplications were detected 2-fold more frequently than microdeletions (39 vs. 18). In 0.9% cases (5/542) the clinical impact of detected CNVs could not be assessed due to either absence of parental DNA samples for confirmatory analysis or relevant literature about CNVs in these chromosome locations.

Among the 68 patients with 74 pathogenic/likely pathogenic CNVs and normal karyotype, 36.5% CNVs (27/74) were de novo in origin, 25.7% CNVs (19/74) were inherited from a healthy or mildly affected parent (14 CNVs of maternal origin and 5 CNVs of paternal origin). In the remaining cases (28/74) at least one parental DNA sample was not available for analysis. On the other hand, 94.7% all the likely benign CNVs (54/57) were inherited and only one had de novo origin. Among 15 cases of VOUS (including the patient with marker chromosome derived from chromosome 21) we identified the de novo origin of 17.6% CNVs (3/17) and 23.5% cases (4/17) of inherited CNVs; in remaining CNVs their origin has not been assessed so far.

**Fig. 2:**
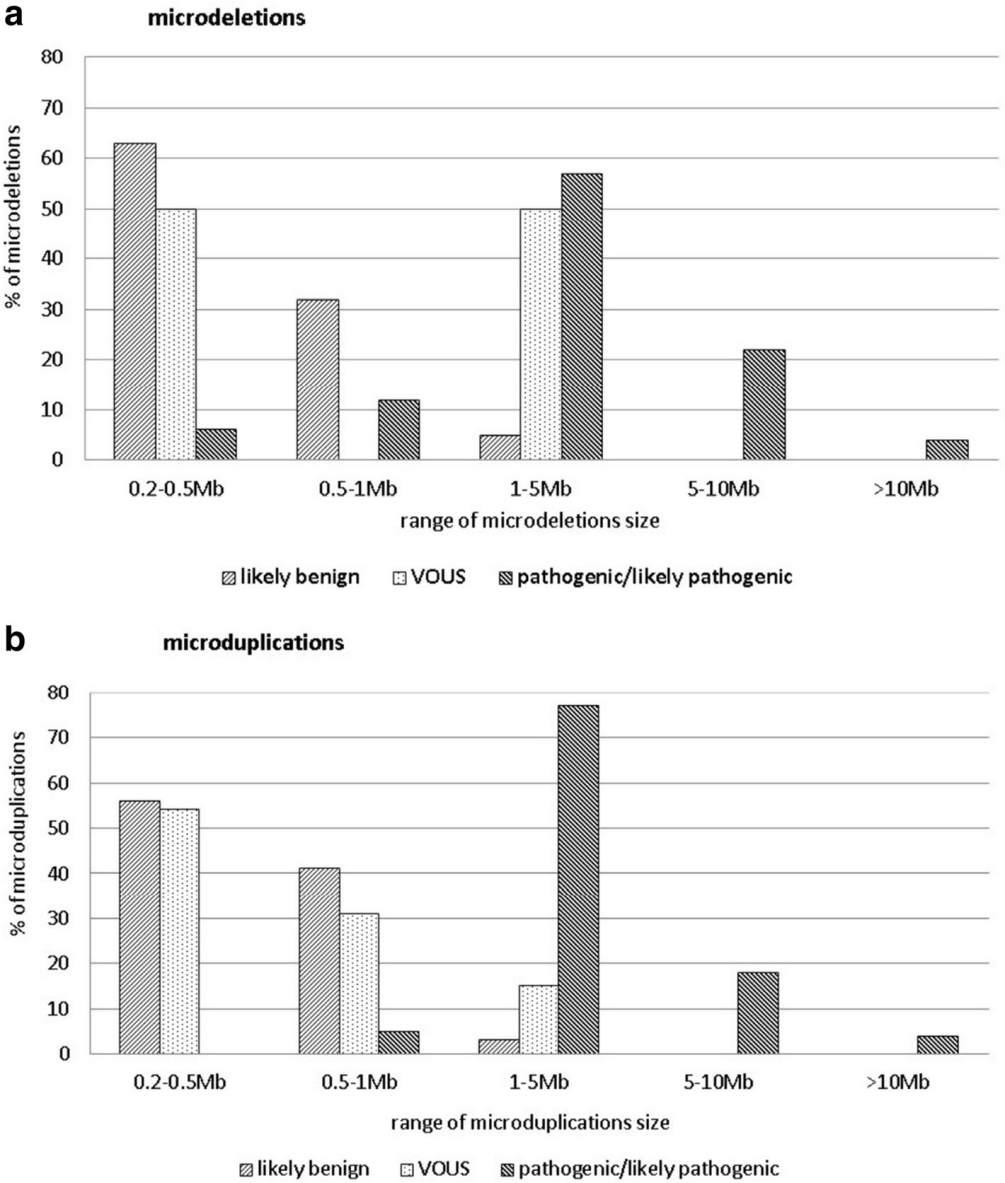
The relationship between the size of submicroscopic CNVs and their clinical impact in children with ID/DD, ASD and MCA – **a**) microdeletions, **b**) microduplications. The data are based on the analysis of 147 CNVs detected in 132 children’s patients with ID/DD, ASD and MCA. The CNVs observed in patients with positive cytogenetic findings and the cases with detected cnnLOH were excluded from this analysis. The sum of CNVs in every classification category is 100%. **a** The relationship between the size of microdeletions and their clinical impact in children’s patients with ID/DD, ASD and MCA. The data are based on the analysis of 147 CNVs detected in 132 children’s patients with ID/DD, ASD and MCA. The CNVs observed in patients with positive cytogenetic findings in karyotype as well as those cases with detected LOH were excluded from this analysis. **b** The relationship between the size of microduplications and their clinical impact in children’s patients with ID/DD, ASD and MCA. The data are based on the analysis of 147 CNVs detected in 132 children’s patients with ID/DD, ASD and MCA. The CNVs observed in patients with positive cytogenetic findings in karyotype as well as those cases with detected LOH were excluded from this analysis

Comparing the size of CNVs to their clinical impact, it is clear that the likely benign CNVs are mostly smaller than 1 Mb. We detected 57 likely benign CNVs (median size 0.394 Mb, range 0.205–1.469 Mb) and only two of them were larger than 1 Mb, but smaller than 1.5 Mb. In cases presenting submicroscopic CNVs of uncertain significance (median size 0.503 Mb, range 0.240–3.238 Mb), 50% CNVs (8/16) were smaller than 0.5 Mb, but we also detected 25% CNVs (4/16) larger than 1 Mb. In contrast among 74 pathogenic or likely pathogenic CNVs detected in 68 patients with normal karyotype we observed that 86% CNVs (64/74) were larger than 1 Mb. Categorizing the CNVs as either microdeletions and microduplications, we can observe the different size distribution between microdeletion and microduplication VOUS as shown in Fig. 2a, b.

## Discussion

The introduction of DNA microarrays has introduced a major advance in the discovery of new submicroscopic rearrangements in the pathogenesis of idiopathic ID/DD and associated congenital abnormalities. This was reflected by a rapidly increased diagnostic yield so it was with little surprise that array-CGH was adopted and recommended as a first-tier clinical diagnostic test for such patients [12, 13]. The discovery of many new microdeletion and microduplication loci across the human genome represents a challenge to determine the pathogenicity and clinical impact introduced by these chromosome rearrangements.

In the course of our 6-year study, we examined 542 children with ID/DD, ASD and MCA by array-CGH using oligonucleotide DNA microarrays. In 32.7% of them (177/542), we detected non-polymorphic CNVs or large regions of cnnLOH with potential clinical relevance. Determining the clinical impact of CNVs is the critical point in array-CGH data interpretation. To this end, we considered their size, gene content, inheritance pattern and information from databases and relevant literature [12, 14]. Overall, we identified the pathogenic/likely pathogenic CNVs in 17.7% patients (96/542) which corresponds to the generally held diagnostic yield of 15–20% based on many multicenter studies [20–23]. Further, 5.9% of all patients had associated karyotypic findings (32/542). Based on multiple studies, classical G-banded karyotyping can identify chromosomal aberrations in about 3–5% of children with ID/DD (excluding Down syndrome and other known chromosome syndromes) [12, 24]. We have shown that array-CGH provides highly reliable confirmatory test specifying the size and gene content of chromosomal aberrations detected by postnatal G-banding karyotype analysis.

In the group of patients with normal karyotype we identified 7.1% (36/510) patients with known recurrent microdeletion and microduplication syndromes and 5.1% (26/510) patients with non-recurrent microdeletions and microduplications. Consequently, we divided the patients sharing recurrent CNVs into small groups. These groups of patients having these single recurrent pathogenic/likely pathogenic CNVs share some notable phenotypic features as ID/DD, ASD and facial stigmatization, however, some phenotypic features are patient-specific. This may indicate the partial impact of multiple loci acting across the human genome as modifiers, or, especially in the cases of microdeletion syndromes, the different expression of alleles present in the hemizygous state [25]. The occurrence of phenotypic modifiers can also explain cases of inherited microduplication syndromes, especially from a healthy parent. These modifiers could be specific for every family and probably lead to incomplete penetrance and variable expressivity of the genes included in the detected CNVs. For example, in our cohort of 36 patients with single recurrent pathogenic/likely pathogenic CNVs we identified three cases of 15q11.2 microdeletion of maternal origin, one case of 15q11.2-q13.1 duplication of paternal origin and one case of maternal origin, one case of 22q11.21 microduplication inherited from a mildly affected mother and one case of 22q11.21 microduplication of paternal origin. In these cases, we can expect the modifying impact of multiple loci across the human genome, which could explain the more severe phenotype in children than in their parents or the variability of phenotypes among children sharing the same CNVs.

Rosenfeld et al. (2013) performed Bayesian computational analysis, which arose from the CNVs frequencies in more than 22,000 controls and more than 48,000 postnatal cases of ID/DD, autism and MCA [26]. They calculated the penetrance for a large variety of CNVs, including all those considered in this article and estimated very low penetrances eg. 10,4% in the case of the 15q11.2 microdeletion. In our cohort, we identified three cases of maternally inherited 15q11.2 microdeletion and in all of them the mother was unaffected or had very mild phenotype. 15q11.2 microdeletion has been recently defined as one of the most common chromosomal abnormality in the pathogenesis of ASD [27]. In contrast we also identified three cases of proximal 16p11.2 microdeletion (two of them of de novo origin). For this CNV the penetrance was calculated as Rosenfeld et al. (2013) as 46.8%, which is compatible with its powerful adverse impact on the phenotype [26]. In our study, it was not surprising that de novo CNVs appeared to have a higher general penetrance. The penetrance analysis also supports the role of other CNVs (proximal 1q21.1 microduplication, 15q11.2 microdeletion, 16p13.11 microdeletion, 16p12.1 microdeletion, distal 16p11.2 microdeletion) with low penetrance (< 20%) as “risk” or “susceptibility loci” in the pathogenesis of ID/DD, ASD and MCA, examples of which are present in our cohort.

Girirajan et al. (2010) presented a two-hit model for cases presenting severe neurodevelopmental delay [28]. They observed a significant proportion of cases with a second large CNV among cases with one specific CNV (16p12.1) compared to controls. They suggested the impact of “susceptibility loci” and the strong effect of a second CNV in the manifestation of severe phenotypic features. When the second CNV is not detected due to the resolution limit of microarray analysis, the approaches of whole-exome (WES) and whole-genome sequencing (WGS) can identify the second hit (rare or de novo DNA sequence variant or small CNV) in cases with the CNVs acting as “risk” or “susceptibility loci” [28, 29].

In our cohort, we can clearly demonstrate the two-hit model in case 451 with a maternally inherited 11p14.3-p15.1 microduplication (6.667 Mb) and de novo microdeletion 10q26.2-q26.3 (6.231 Mb). The terminal 10q26.2-q26.3 microdeletion is already referred as the “Chromosome 10q26 deletion syndrome” (OMIM #609625). Despite the presence of a 11p14.3-p15.1 microduplication (outside the Beckwith-Wiedemann critical region) the mother did not exhibit apparent phenotypic features, so we consider the presence of positively modifying effect of combined multiple loci across her genome and/or the absence of a second pathogenic hit as the most likely explanation.

Besides recurrent pathogenic/likely pathogenic CNVs, we detected a group of unique non-recurrent pathogenic/likely pathogenic CNVs. We analyzed them for their gene content to identify any candidate genes which could support their pathogenicity. Subsequently we estimated their origin in cases when parental DNA samples were available (see Additional file 3). As example, in patient 490 we detected 6q15-q161.1 microdeletion (2.141 Mb) which affected the *EPHA7* gene. Based on data from OMIM database (www.omim.org), this gene has not been clearly related to any congenital genetic disease so far. It encodes the ephrin type-A receptor of protein-tyrosine kinase family. These receptors have been implicated in mediating development of the central nervous system [30]. Its role as a candidate gene in neurodevelopmental disorders is supported by a case of a 6q16.1 microdeletion in a child with neurological abnormalities [31].

In our cohort of patients with pathogenic/likely pathogenic submicroscopic CNVs we observed more than 2-fold higher frequency of microdeletions than microduplications (70% vs. 30%). Multiple studies proved the higher likehood of pathogenicity for microdeletions than for their reciprocal microduplications due to the content of dosage-sensitive haploinsufficient genes, then these microduplications usually cause milder phenotype [32, 33]. The level of pathogenicity of those CNVs can be also modified by the incomplete penetrance or by the position effect in the case of microduplications. In general, the vast majority of microduplications occur in tandem in orientation and are often inherited from healthy or slightly affected parent. However, the microduplication larger than 1 Mb and located elsewhere in the genome, are expected to be likely pathogenic. For the clinical interpretation of microduplication it is generally recommended to assess their origin, to analyse the gene content and to search public databases of CNVs, and if possible, to locate them in the genome [34].

Concerning CNV size, we identified that CNVs larger than 1 Mb are more likely to have pathogenic/likely pathogenic effect on phenotype. We also showed that vast majority of CNVs smaller than 500 kb had familial origin as reviewed in Miller et al. (2010) [12]. In detail, we detected a total of 46 CNVs smaller than 500 kb with defined clinical impact: 76% of them had familial origin (35/46), 4% of them were de novo of origin (2/46) and 20% of them (9/46) have not assessed their origin so far.

Miller et al. (2010) also reviewed extensive studies based on HapMap samples, concluding that 90–95% of likely benign CNVs were smaller than 500 kb. Based on the synthesis of multiple array-CGH analyses they recommend a resolution ≥400 kb for CNVs as a general analytical and clinical threshold. All these facts in data interpretation are necessary to evaluate every CNV independently in the context of the patient’s phenotype, CNV origin, size, gene content and published information obtained from databases and literature. To demonstrate the CNV size as only one of more criteria to specify its clinical impact, we identified 58% of likely benign CNVs (33/57) smaller than 500 kb, but on the other hand also three cases of known susceptibility regions for neurodevelopmental disorders (two cases of maternally derived 15q11.2 microdeletion and one case of 16p12.2 microdeletion of unknown origin) of size smaller than 500 kb.

Using 180 K CGH + SNP microarray platform we detected 1.5% cases (8/542) with large stretches of cnnLOH > 5 Mb, in which we identified from tens to hundreds of genes related to autosomal recessive disorders. The presence of more than one LOH locus indicates the parental consanguinity which is typical for individuals from inbred populations [35, 36]. Based on low incidence of consanguinity in the Czech Republic (~ 0.2%) (www.consang.net) [37] we suggest to use CGH + SNP microarray platforms for analysis in specific families when the parental consanguinity is reported in the preliminary genetic counselling. As another step in molecular diagnostics algorithm, the approaches of targeted Sanger sequencing, WES or WGS can identify the particular pathogenic DNA sequence variants with autosomal recessive inheritance in these families [38, 39].

In the course of the study we used several types of DNA microarrays (180 K and 60 K) but were unable to see any differences in their diagnostic reliability. So, although 180 K platforms offer a 3-fold higher resolution, we can also recommend the 60 K platforms for routine clinical array-CGH analyses, as shown by Sansović et al. (2017) [23]. However, to confirm the complementarity and specificity of different microarray platforms we suggest reanalyses of DNA samples (with any detected CNVs) once or twice a year as an internal quality check for diagnostic laboratories. Based on the newly published recommendation, the microarray platform should achieve a resolution at least 200–400 kb for postnatal analyses, so both 60 K and 180 K microarray platforms are fully sufficient to comply this requirement [12, 39, 40].

Following banding based karyotyping, array-CGH entered the field of molecular cytogenetic analyses and immediately significantly improved the diagnostic yield [41]. However, despite its high sensitivity, accuracy and reliability, array-CGH cannot entirely replace the standard conventional G-banding karyotyping, due to its inability to detect balanced chromosomal rearrangements or to specify gains of particular DNA sequences in the karyotype. In some patients we proved the significance of the combined strategy including G-banding karyotyping and array-CGH. G-banding karyotyping facilities the global and complex analysis of chromosomal organization and array-CGH confirms and specifies the chromosomal rearrangements on molecular level.

Besides being a well-established routine test for the rapid detection of suspected microdeletion syndromes, FISH analysis can identify the position of duplicated loci in microduplication syndromes. Using specific locus probes, it is possible to identify cryptic balanced inversions in parents, resulting in CNVs arising in the offspring following meiotic recombination within the inversion segment of the carrier parent [42–44]. Thus, due to the wide range in its diagnostic capacity, array-CGH is a standard method in routine diagnostic multistep algorithm, where it serves as a first-tier test, confirmatory test or test following conventional G-banding karyotyping as well. Although our microarray studies have demonstrated the importance of de novo CNVs in ID/DD related disorders, the majority of our cases remain undiagnosed. Recent studies showed that WES/WGS can be applied for simultaneous detection and characterization of both CNVs and single-nucleotide variants, which could reduce the number of analyses to one complex test to reach the diagnosis [45]. In our pilot study using targeted NGS with commercially available gene panel we identified pathogenic sequence variants in some patients with negative array-CGH assay and confirm the diagnosis on the molecular level [46].

## Conclusions

Our study confirms array-CGH as an effective diagnostic tool for both detection and precise characterization of clinically important CNVs in children with ID/DD, ASD and MCA. Due to its wide application and clear cost effectiveness, array-CGH now offers the most efficient cytogenetic screening method routinely used in clinical laboratories and it is only likely to be replaced by when the costs of NGS are reduced by an order of magnitude.

## Additional files

Additional file 1: Confirmation of chromosomal aberrations detected by G-banding karyotype in 32 children with ID/DD, ASD and MCA. (XLSX 12 kb)

Additional file 2: Recurrent pathogenic/likely pathogenic CNVs detected by array-CGH in 36 children with ID/DD, ASD and MCA. (XLSX 14 kb)

Additional file 3: Non-recurrent pathogenic/likely pathogenic CNVs detected by array-CGH in 26 children with ID/DD, ASD and MCA. (XLSX 14 kb)

Additional file 4: Children’s patients with two pathogenic/likely pathogenic CNVs. (XLSX 11 kb)

Additional file 5: CNVs of uncertain significance detected by array-CGH in 15 children with ID/DD, ASD and MCA (List 1), cnnLOH detected by CGH + SNP microarrays in 8 children with ID/DD, ASD and MCA (List 2). (XLSX 17 kb)

Additional file 6: Likely benign CNVs detected by array-CGH in 50 children with ID/DD, ASD and MCA. (XLSX 14 kb)

## Data Availability

Microarray data are available in the ArrayExpress database (www.ebi.ac.uk/arrayexpress) under accession numbers E-MTAB-6685, E-MTAB-6733, E-MTAB-6731, E-MTAB-6734, E-MTAB-6735 [47]. The other supporting data, i.e. data from cytogenetic analyses of karyotype (G-banding) and confirmatory analyses (FISH, MLPA, relative qPCR) are available from the corresponding author on the reasonable request.

## Abbreviations

array-CGH: array-based comparative genomic hybridization
ASD: Autism spectrum disorders
cnnLOH: copy-number neutral loss of heterozygosity
CNV: Copy number variant
CNVs: Copy number variants
FISH: Fluorescence in situ hybridization
FoSTeS: Fork stalling and template switching
ID/DD: Intellectual disability and developmental delay
MCA: Multiple congenital anomalies
MLPA: Multiplex ligation-dependent probe amplification
NAHR: Non-allelic homologous recombination
NGS: “Next-generation” sequencing
NHEJ: Non-homologous end joining
qPCR: quantitative polymerase chain reaction
VOUS: Variant of uncertain significance
WES: Whole-exome sequencing
WGS: Whole-genome sequencing
